# Assessing the impact of pregnancy and birth factors on the maternal and infant microbiota

**DOI:** 10.20517/mrr.2023.32

**Published:** 2023-07-24

**Authors:** Kait F Al, Laura Allen, Samantha Bedell, Jeremy P Burton, Barbra de Vrijer

**Affiliations:** ^1^Canadian Centre for Human Microbiome and Probiotic Research, Lawson Health Research Institute, London, Ontario N6A4V2, Canada.; ^2^Department of Microbiology and Immunology, Western University, London, Ontario N6A3K7, Canada.; ^3^London Health Sciences Centre, London, Ontario N6A5W9, Canada.; ^4^Department of Obstetrics and Gynaecology, Division of Maternal Fetal Medicine, Western University, London, Ontario N6H5W9, Canada.; ^5^Division of Urology, Department of Surgery, Western University, London, Ontario N6A4V2, Canada.; ^6^Children’s Health Research Institute, London, Ontario N6C 4V3, Canada.

**Keywords:** Microbiome, microbiota, maternal, infant, antibiotics, Group B streptococci, obesity, perinatal factors

## Abstract

**Background:** The microbiota acquired at birth is known to play an intimate role in later life health and disease and has been shown to be affected by the mode of birth. There has been recent interest in microbiota correction by maternal vaginal seeding in Cesarean section-born infants; however, the safety of this practice has been debated. The aim of this study was to assess how other factors, such as timing of sampling, maternal obesity, vaginal Group B *Streptococcus* colonization (GBS), and antibiotic exposure, affect the maternal and infant microbiota.

**Methods:** Maternal vaginal and saliva samples were collected at three time periods: 35-37 weeks gestation (prenatal), within 24-36 hours after birth (birth), and at ~6 weeks postpartum. Infant saliva and stool samples were collected at ~6 weeks postpartum. 16S rRNA amplicon sequencing was utilized to assess the taxonomic and inferred functional compositions of the bacterial communities from both mothers and infants.

**Results:** Samples from 36 mothers and 32 infants were obtained. Gestational age, breastfeeding, mode of birth, and gravidity were associated with taxonomic alterations in the infant samples, while obesity, antibiotic use, and GBS status were not. Maternal samples were predominantly affected by time, whereby significant alterations including increased microbial diversity were seen at birth and persisted to 6 weeks postpartum.

**Conclusion:** This study provides information on the relationship between health and delivery factors and changes in vaginal and infant microbiota. These results may better direct clinicians and mothers in optimizing the infant microbiota towards health during infancy and later life.

## INTRODUCTION

The human body harbors abundant microbiota, which varies by anatomical site and influences health and disease, including metabolic, immune, and infectious conditions^[1]^. The composition of a newborn’s microbiota is altered by a variety of host genetic and environmental factors, and primary colonization occurs at birth by vertical transmission from the mother. During pregnancy, the vaginal microbiota is important for the health of both mother and fetus, and changes in vaginal microbiota are associated with pregnancy complications such as preterm birth and late miscarriage^[2]^.

The mode of birth has been shown to be a major determinant of infant microbiota composition with potentially extensive later-life health implications^[3]^. A vaginal birth exposes the baby to maternal vaginal and fecal bacteria, while Cesarean section-born infants are enriched in skin-associated bacteria^[3,4]^. After birth, Cesarean section microbial acquisition in the infant has been associated with an increased prevalence of chronic immune conditions^[5]^. There has been recent interest in the practice of exposing infants to maternal vaginal fluids immediately following a Cesarean birth, commonly known as vaginal seeding. However, this practice does not consider changes in maternal microbiota, potentially putting infants at an increased risk of infection, and the long-term health consequences of vaginal seeding have yet to be established^[6]^.

In addition to the mode of birth, factors such as maternal body mass index (BMI), antibiotic use during pregnancy, and early-life antibiotic use in the infant are also believed to affect normal microbiota acquisition^[7-9]^. Obesity rates have been steadily increasing and affect women of reproductive age, with approximately 45% beginning their pregnancy either overweight or obese^[9]^. Obesity, regardless of and in the case of pregnancy, affects the composition of the microbiota^[7,10]^ and is associated with higher rates of antibiotic use^[11]^. Obesity during pregnancy is also associated with longer labors^[12]^, higher rates of Cesarean section^[13]^, chorioamnionitis^[14]^, and Group B *Streptococcus* (GBS) colonization^[15,16]^, which in North America is managed with routine antibiotic administration during labor^[17]^, all factors that may independently affect the acquisition of a normal microbiome. Furthermore, breastfeeding success rates are lower in women who have delivered by Cesarean section and in women with obesity regardless of mode of delivery^[18,19]^. Importantly, with few exceptions, most pregnancy-related microbiota studies have focused on normal weight mothers or do not follow the infant microbiota, and thus the impact of other pregnancy factors remains unclear^[20-22]^.

Through an assessment of taxonomic and inferred functional alterations in the vaginal and salivary microbiota from mothers, as well as salivary and gut microbiota from infants, this study sought to highlight the correlations between pregnancy and delivery factors, such as obesity, GBS status, antibiotic use, and breastfeeding, as they relate to changes in maternal and infant microbiota. This information may better direct clinicians and mothers towards optimizing the infant microbiota for later life health.

## METHODS

### Study design and sample collection

This study was approved by the Health Sciences Research Ethics Board (HSREB# 105630) and written informed consent was received from all participants. Individuals with singleton pregnancies were recruited from London Health Sciences Centre, Victoria Hospital in London, Ontario, Canada. Individuals who received antibiotics in labor for presumed chorioamnionitis or prior to labor for conditions affecting the immune system (e.g., auto-immune disease, severe anemia, cardiac conditions, and severe preeclampsia) were under the age of 18 years or over the age of 40 years, or had a fetus with suspected congenital anomalies, severe growth restriction or chromosomal disorders were excluded from the study. Data such as maternal age, height, pre-pregnancy weight, and gestational weight gain, as well as infant birth weight and type of feeding were collected.

Demographic information, GBS screen results, and pregnancy and birth data were obtained from patient charts. An Upset plot was generated in R (version 4.2.1) with the UpSetR package (version 1.4.0) to display the intersection of study participants based on eight birth factors. Additionally, maternal vaginal and saliva swab samples were collected during three perinatal periods: around 35-37 weeks gestation (prenatal), within 24-36 hours after birth (birth), and at ~6 weeks postpartum (postpartum). Infant saliva and stool swabs were collected at the postpartum appointment approximately 6-8 weeks after birth. All swabs were stored at -80 °C until downstream processing.

### Bacterial DNA isolation, amplification, and sequencing

Swabs were cut directly into the wells of the bead plate from a 96-well DNeasy PowerSoil HTP 96 kit (QIAGEN, Toronto, ON) and the protocol was followed as per the manufacturer’s directions, as previously described^[23]^. A Biomek® 3000 Laboratory Automation Workstation was utilized for automated PCR reagent set-up^[24]^. Amplifications of the V4 region of the 16S ribosomal RNA gene were carried out with the primers (5'-3') ACACTCTTTCCCTACACGACGCTCTTCCGATCTNNNNxxxxxxxxxxxxGTGCCAGCMGCCGCGGTAA and (5'-3') CGGTCTCGGCATTCCTGCTGAACCGCTCTTCCGATCTNNNNxxxxxxxxxxxxGGACTACHVGGGTWTCT wherein "xxxxxxxxxxxx" represents a sample-specific nucleotide barcode and the preceding sequence is a portion of the Illumina adapter sequence for library construction. The Biomek® robot transferred 2 μL of the DNA template into a plate containing 10 μL of forward and reverse primers (3.2 pmol/µL). Then 20 μL of Promega GoTaq® Colourless Master Mix (Promega, Maddison, WI) was added to the DNA template and primers. The final plate was firmly sealed with a foil PCR plate cover. This plate was placed in the thermal cycler (Techne, Mississauga, ON) where the lid was kept at 105 °C. An initial warm-up temperature of 95 °C was used for 2 min to activate the GoTaq®. Afterwards, the volumes underwent 25 cycles of 95 °C for 1 min, 52 °C for 1 min, and 72 °C for 1 min. After completion, the temperature of the thermal cycler was held at 4 °C and amplicons were then stored at -20 °C.

DNA sequencing was conducted at the London Regional Genomics Centre at Robarts Research Institute (London, ON). Amplicons were quantified using PicoGreen (Quant-It; Life Technologies, Burlington, ON) and pooled at equimolar concentrations before cleanup (QIAquick PCR clean up; QIAGEN inc). The final samples were sequenced using the MiSeq by Illumina® platform, with 2 × 260 bp paired-end chemistry with a cluster density of 1100 and 5% PhiX.

### Sequencing dataset processing

After sequencing, the paired reads were demultiplexed using custom scripts, then quality filtered, trimmed, denoised, and merged following the DADA2 pipeline (version 1.26.0)^[25]^ in R. The SILVA Database (version 138) was utilized in assigning taxonomy to the amplicon sequence variants (SVs)^[26]^. Further filtering was performed such that SVs were removed that did not comprise > 1% of the relative abundance in any sample.

Inferencing of microbial metagenomes was performed with PICRUSt2 (version 2.4.1)^[27]^. Downstream analysis was performed on both gene families (Enzyme classification numbers, EC) and functional pathways (BioCyc ID). Gene family and pathway counts were not filtered but were rounded to integers for downstream analyses.

### Statistical analyses

Alpha diversity (Shannon’s Index) was calculated using custom R scripts. A Kruskall-Wallis test followed by a Dunn’s multiple comparison test (from the rstatix R package, version 0.7.2) was used to test for alpha diversity differences between sample subgroups. Sparsity in read count data was zero-adjusted with the cmultRepl function from the zCompositions R package (version 1.4.0-1)^[28]^. The zero-adjusted counts underwent centered log ratio (CLR) transformation and a principal components analysis (PCA) was performed. PCA biplots were generated with ggplot2 (version 3.4.2)^[29]^. Sample type comparisons of Aitchison distance (β-diversity) were calculated by PERMANOVA with the adonis2 function from the vegan R package (version 2.6-4)^[30]^. Potential covariates of microbiota composition were determined using the envfit function from the vegan R package with the Aitchison distance PCA as the input object. Differential abundance tests of taxonomy, inferred gene families, and functional pathways were determined using a generalized linear mixed model approach with the MaAsLin2 R package (version 1.12.0)^[31]^. Briefly, CLR-transformed counts were used as the input, and the fixed effects were potential covariate clinical metadata features with an envfit *P*-value < 0.1. For maternal samples, the longitudinal repeated measures were accounted for by treating the ‘participant’ as a random effect. For all differential abundance analyses, Benjamini-Hochberg adjusted *P*-values (*Q*-values) < 0.1 were considered significant. Co-occurrence network analysis was performed using CoNet software (version 1.1.1), as previously described^[32,33]^, where only significant (Benjamini-Hochberg corrected Browns *P*-value < 0.05) edges were plotted. CoNet networks were visualized in Cytoscape (version 3.8.2)^[34]^, then the final copresence and mutual exclusion network figures were generated with Circos (version 0.69-9)^[35]^. Relative network positivity was calculated as the number of copresence edges divided by the number of mutual exclusion edges. Network property and baby stool microbiota origin figures were generated with GraphPad Prism (version 9.5.1).

## RESULTS

**Figure 1:**
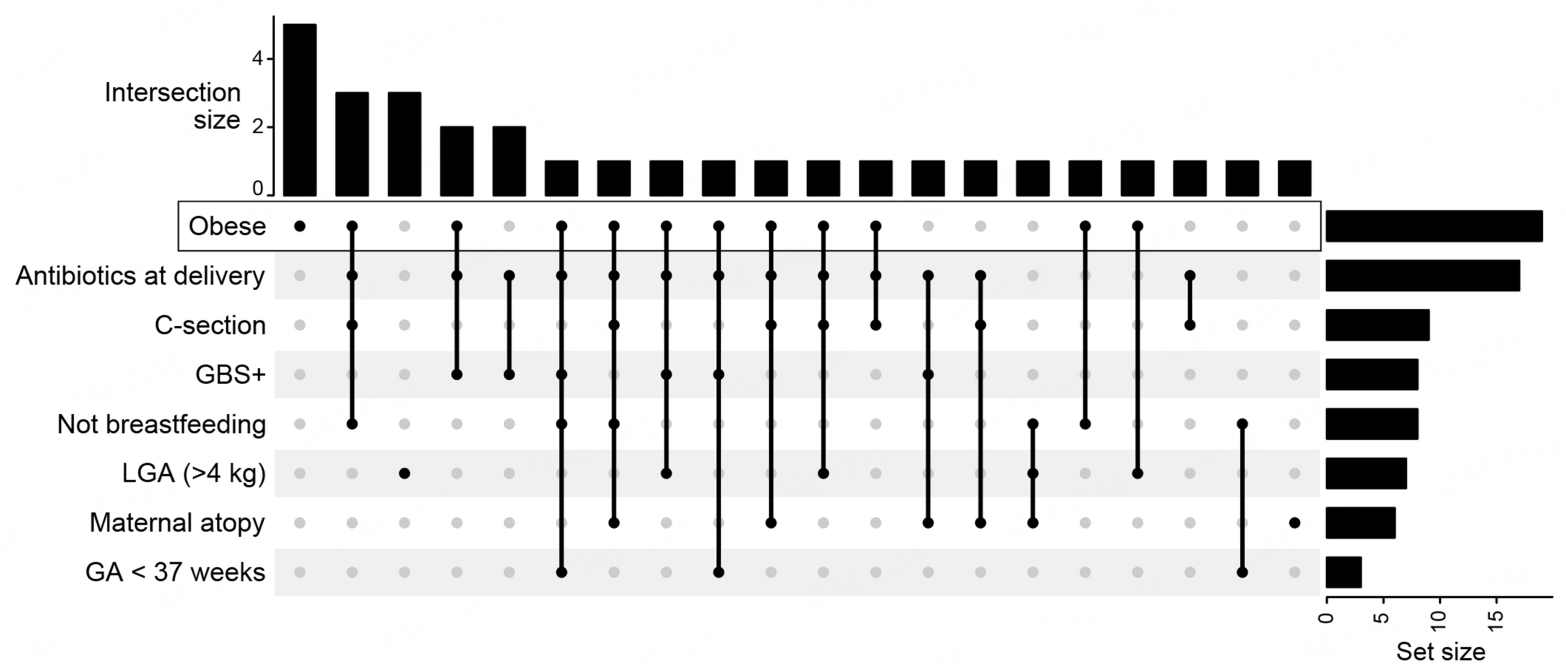
Adverse birth factors are concomitant. Upset plot depicting the intersecting study participants between eight birth factors. Each column corresponds to a factor or set of factors. The number of participants within each set (Intersection size) is displayed above the column. Set size represents the number of participants fitting the specified binary factor (row). Mothers with BMI ≥ 30 were considered obese. LGA: large for gestational age; GA: gestational age.

A total of 36 mothers and 32 infants were recruited for this study. Patient demographic characteristics are summarized in Table 1. Briefly, the cohort was heterogeneous with a range of maternal age, BMI, antibiotic exposure, delivery method, infant feeding, and other aspects. Maternal obesity was associated with higher rates of Cesarean birth, GBS, and antibiotic use during the perinatal or postpartum period, as well as lower breastfeeding rates. [Figure 1].

**Table 1:** Clinical characteristics of study participants

**Patient Characteristics**	**Mean ( ± SD) or *n* (%)**
Age at delivery (years)	31.2 ± 5.5 (range 21-46)
Pre-pregnancy BMI	33.4 ± 10.6 (range 21-49)
BMI ≥ 30	20 (56)
Gravidity	2.4 ± 1.4 (range 1-7)
Primigravida	8 (22)
Gestational age at birth (weeks)	38.9 ± 1.2 (range 35.6-40.7)
Mode of delivery	
Vaginal	27 (75)
Cesarean section	9 (25)
GBS positive	9 (25)
Antibiotic exposure	
During delivery	18 (50)
Postpartum	2 (6)
Infant sex	
Female	17 (47)
Male	19 (53)
Infant birth weight (g)	3498 ± 476 (range 2300-4370)
Infant birth length (cm)	51.8 ± 2.9 (range 46-58)
Breastfeeding	24 (67)

**Figure 2:**
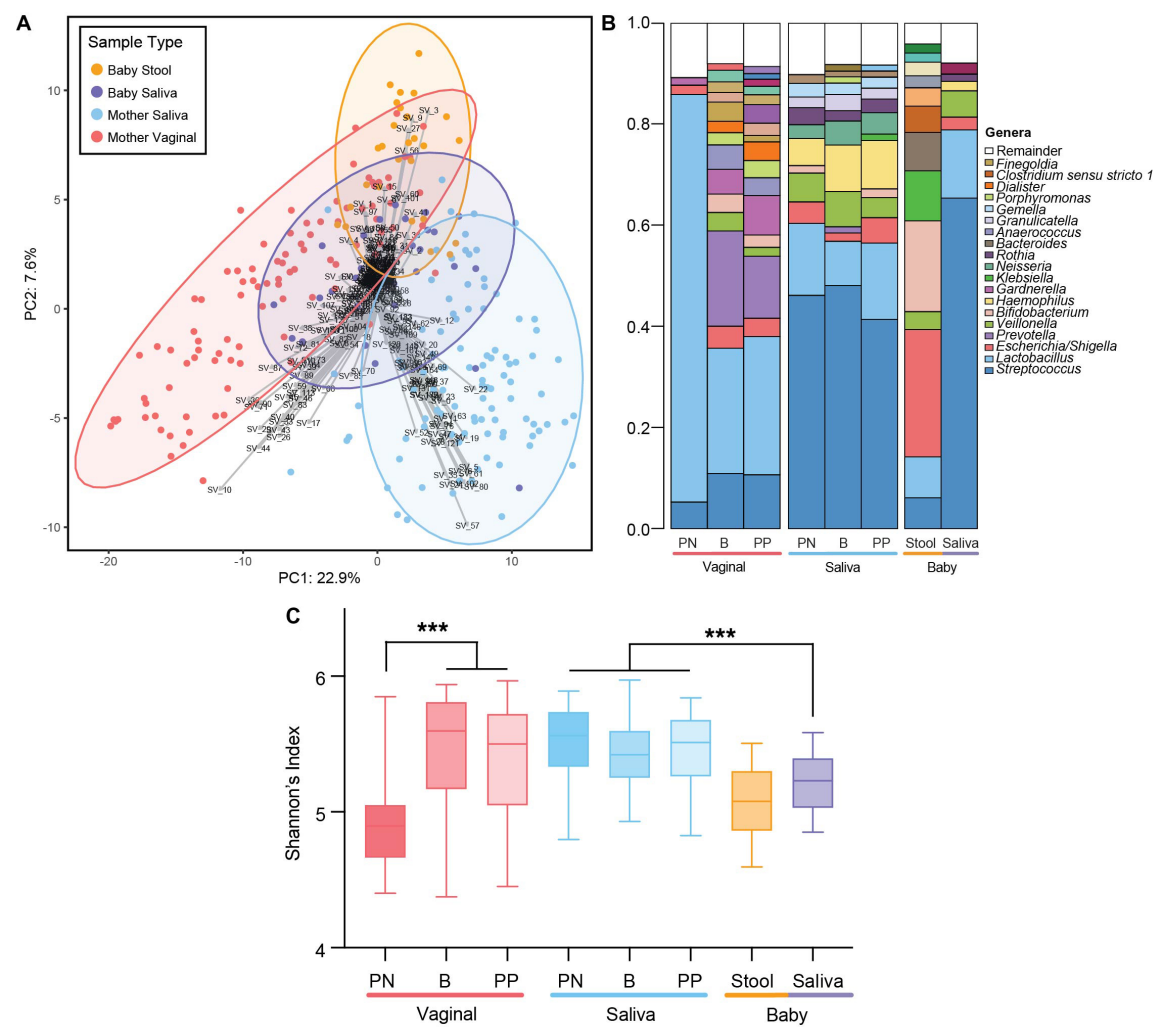
Microbiota composition diverges by sample type and perinatal period. (A) PCA biplot based on sample-wise CLR-transformed Aitchison distance. Each point represents a sample. Strength and association for SVs are depicted by the length and direction of the gray arrows, respectively. Samples are colored by type and ellipses represent 95% confidence intervals. (B) Average relative genus abundance by sample type and perinatal period. Each vertical bar represents the average relative abundance within the sample type subgroup, colored by genus. Genera representing < 1% relative abundance were grouped into the “Remainder” portion. (C) Alpha diversity plot displaying the Shannon index for maternal and infant samples at different perinatal periods. Box plot whiskers represent minimum and maximum. PN: Prenatal; B: Birth; PP: Postpartum; CLR: centered log ratio; PCA: principal components analysis; SVs: sequence variants.

16S rRNA gene sequencing was performed to elucidate the potential impacts of birth and delivery factors on the maternal and infant microbiota. In total, 256 samples were sequenced from the 68 individuals (36 mothers and 32 infants). Following quality filtering with DADA2, the 16S dataset yielded 11,820,704 reads; the average sample read depth was 46,175 (range 2499 -202,456). 1341 unique SVs were initially identified, with 271 SVs remaining after filtering out those that did not comprise > 1% of the relative abundance. For the principal component analysis (PCA) of all samples, 30.5% of the total variance was explained by the first two principal components [Figure 2A]. The samples clustered based on type, with maternal saliva, maternal vaginal, infant saliva, and infant stool samples being significantly distinct (PERMANOVA r^2^ = 0.159, *P* < 0.001; Figure 2A and B). Shannon’s index of alpha diversity also significantly differed between sample groups (Kruskal-Wallis test, *P* < 0.0001). The diversity of baby saliva was significantly lower than the mothers’ (Dunn’s pairwise tests, adjusted *P* < 0.001). Vaginal diversity was lowest in the prenatal samples and was significantly increased following birth and in the postpartum samples (Dunn’s pairwise tests, adjusted *P* < 0.0001; Figure 2C).

**Figure 3:**
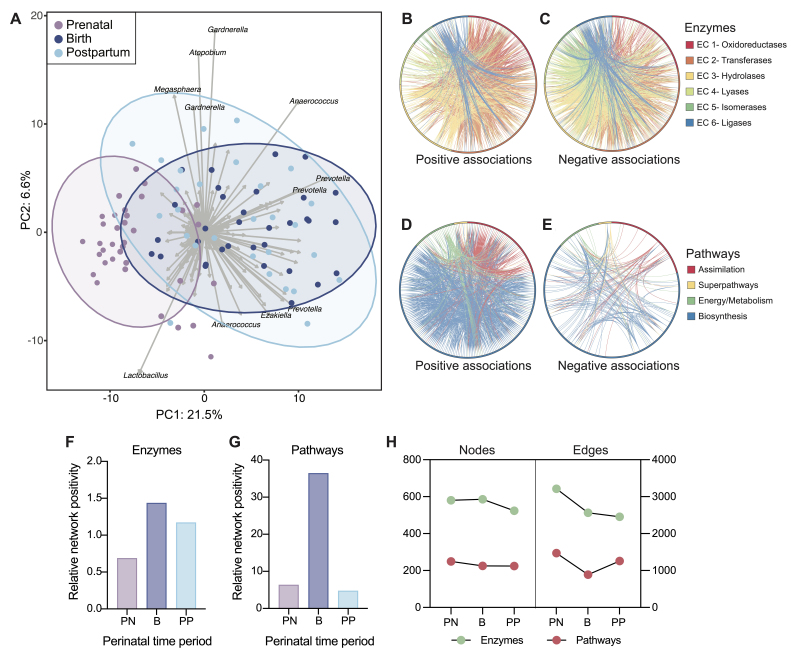
Taxonomic composition and functional co-occurrence network structure are altered in vaginal samples over time. (A) PCA biplot based on sample-wise CLR-transformed Aitchison distance. Each point represents a vaginal sample. Strength and association for genera (SVs) are depicted by the length and direction of the gray arrows, respectively. Samples are colored by perinatal period and ellipses represent 95% confidence intervals. Plots of networks indicate (B) copresence and (C) mutual exclusion patterns of enzymes, and (D) copresence and (E) mutual exclusion patterns of functional pathways in prenatal vaginal samples. Only significant edges are plotted. Edges are colored based on the enzyme or pathway group with more total edges (of the two involved in the interaction). Relative network positivity at both the enzymatic (F) and pathway (G) levels was calculated as the number of copresence edges divided by the number of mutual exclusion edges for the network from vaginal samples at each perinatal period. (H) Total number of nodes and significant edges are plotted for vaginal samples at each perinatal period.

Potential covariates (envfit *P* < 0.1, Supplementary Table 1] were accounted for and sample type subgroups were explored for trends based on clinical features in a multivariate manner [Supplementary Tables 1 and 2]. Perinatal period was the most significant covariate associated with taxonomic and functional changes in the maternal vaginal samples (*P* < 0.001), though BMI and gravidity were also significant factors. Vaginal samples were dominated by lactobacilli prenatally [Figure 2B, Figure 3A, Supplementary Table 2], whereas samples from the birth and postpartum periods were significantly altered in 74 SVs and 36 genera when SV counts were aggregated by genus [Supplementary Table 2]. These postpartum alterations included relative enrichment of the genera *Dialister*, *Prevotella*, *Escherichia*, and *Streptococcus*. BMI was negatively correlated with *Gemella* and *Megasphaera*, while primigravida was associated with a relative depletion of *Escherichia*.

Over 1000 gene families/enzymes and 275 functional pathways were significantly altered in vaginal samples based on perinatal period, gravidity, and BMI [Supplementary Tables 3 and 4]. Co-occurrence network analysis was performed on the vaginal samples’ functional potential at both the gene family and pathway levels [Figure 3B-E]. At both levels, the network structure was significantly altered and became less robust over time, with the relative edge positivity increasing (copresence / mutual exclusion relationships) and the number of significant edges decreasing [Figure 3F-H].

Delivery method and infant sex were determined to be potential covariates of baby stool microbiota. After accounting for these factors, the genus *Citrobacter* was relatively increased in infants delivered by Cesarean section (*Q*-value = 0.04). In baby saliva, the covariates of breastfeeding, gestational age and primigravida were adjusted; infants born prior to 37 weeks harbored relatively more SV41: *Limosilactobacillus fermentum* than those born after 37 weeks (*Q*-value = 1 × 10^-7^), while infants born out of primigravid pregnancies harbored relatively less of the genus *Escherichia* (*Q*-value = 0.048), SV3: *Escherichia* (*Q*-value = 0.05), and SV14: *Bacteroides* (*Q*-value = 0.05) compared to infants of multigravidae.

When investigating microbiota function, baby stool was significantly shaped by the delivery method, whereby infants delivered vaginally were relatively depleted in 41 different gene families compared to those delivered by Cesarean section [Supplementary Table 3]. Several of these enzymes are involved in lipopolysaccharide (EC.2.4.1.58) or *E. coli* O-polysaccharide biosynthesis (EC.2.1.1.294, EC.2.7.1.181). Infants delivered vaginally were also relatively depleted in 13 functional pathways compared to those delivered by Cesarean section [Supplementary Table 4]. Several of these functional pathways were Pseudomonadota-specific, including 3-phenylpropanoate degradation (PWY0.1277) and sulfoquinovose degradation (PWY.7446).

Baby salivary microbiota function was significantly shaped by gravidity, gestational age, and breastfeeding [Supplementary Tables 3 and 4]. For example, breastfed infants had a relatively lower abundance of EC.1.6.3.4, an NADH oxidase enzyme likely originating from *Streptococcus mutans* (*Q* = 0.009), and infants from primigravidae had a relatively lower abundance of the functional pathway for biosynthesis of the virulence factor enterobactin^[36]^ (*Q* = 0.005).

**Figure 4:**
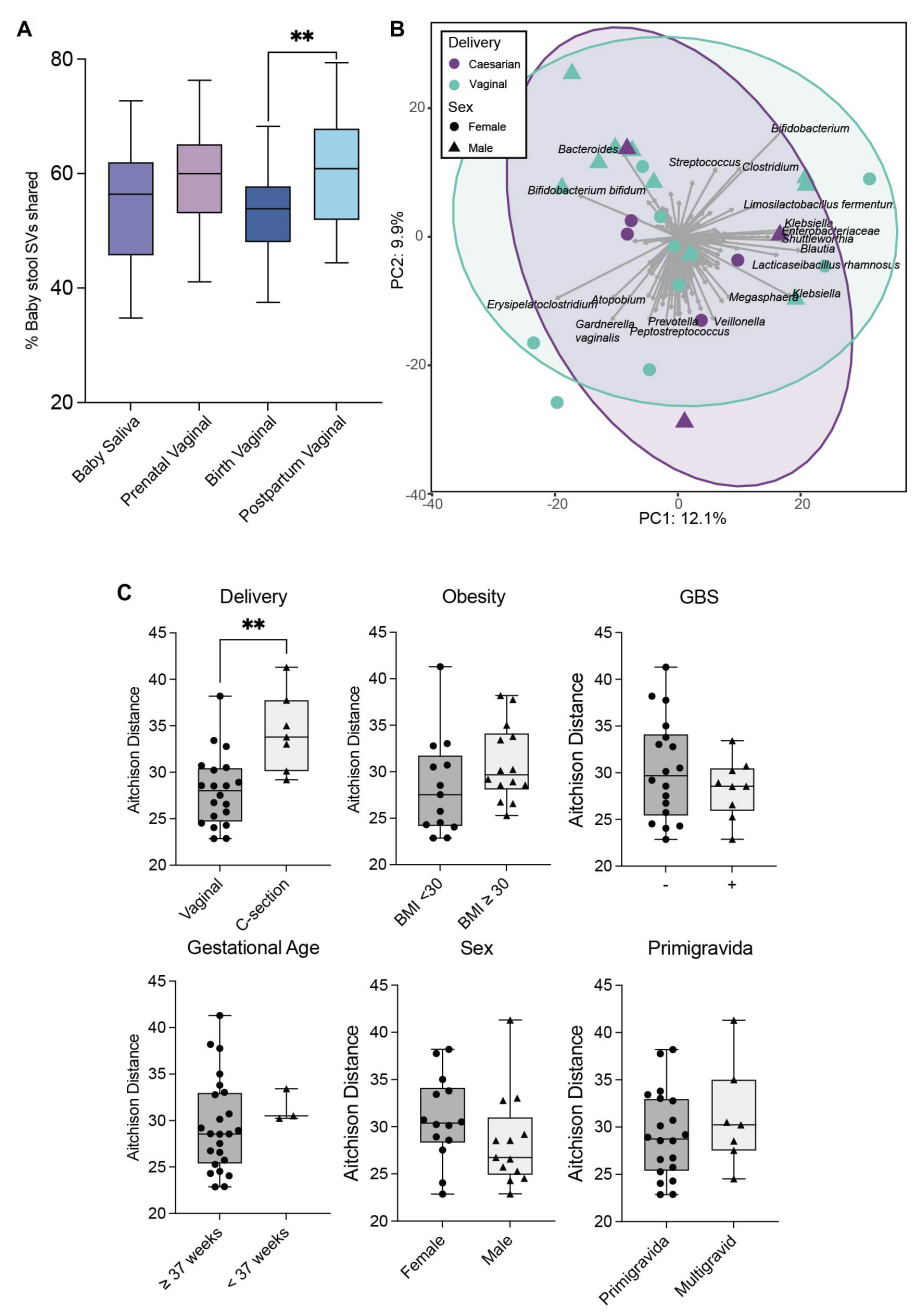
Towards prediction of the origin of baby stool microbiota by dyad analysis. (A) The percentage of baby stool microbiota SVs shared between the same baby’s saliva samples, or their maternal vaginal samples. Box plot whiskers represent the minimum and maximum. (B) PCA biplot based on sample-wise CLR-transformed Aitchison distance. Each point represents a baby stool sample. Strength and association for genera (SVs) are depicted by the length and direction of the gray arrows, respectively. Samples are colored by delivery method and shaped by sex; ellipses represent the 95% confidence intervals of the delivery method. (C) Beta diversity (Aitchison distance) between baby stool samples and prenatal vaginal samples compared based on clinical factors. CLR: centered log ratio; PCA: principal components analysis; SVs: sequence variants.

To track the potential source of baby stool taxa, dyad analysis was performed by determining shared SVs between i) baby stool and baby oral microbiota, and ii) baby stool and vaginal microbiota. This analysis was performed on the filtered SV counts table, so all non-zero SV counts in dyad samples were considered shared. On average, baby stool samples contained 54 SVs, of which 54%, 58%, 53%, and 60% were shared with the baby’s saliva, prenatal vaginal, birth vaginal, and postpartum vaginal samples, respectively (Tukey’s multiple comparison test, *P* = 0.007; Figure 4A). The shared taxa were generally of high abundance (for example, *Lactobacillus iners*, *Lactobacillus crispatus, Lactobacillus jensenii*, and *Streptococcus spp*.), whereas more rare taxa were not consistently shared.

Beta diversity (Aitchison distance) between the baby fecal and prenatal vaginal sample pairs was investigated as a marker of vertical transmission and microbiota engraftment. In these comparisons, the shorter the Aitchison distance, the more similar the baby fecal and prenatal vaginal sample microbiota, suggesting increased “engraftment” from the vaginal microbiota into the baby’s gut. This “engraftment” was compared between different birth factors to explore which, if any, influenced the diversity between the sample dyads. Vaginal delivery led to significantly more engraftment (Bonferroni corrected T-test, *P* = 0.008), as the Aitchison distance between the baby gut microbiota and prenatal vaginal samples was lower than the distance between dyads with Cesarean delivery [Figure 4B and C]. Trends were also observed with more engraftment in male babies and those born to non-obese mothers, although these did not reach statistical significance.

## DISCUSSION

This study investigated how the maternal and infant microbiota are shaped by pregnancy and delivery factors including obesity, timing of sampling, GBS status, antibiotic use, and breastfeeding. It was demonstrated that the vaginal microbiota was significantly altered at the time of birth, regardless of the mode of birth. Other birth factors were less influential but still associated with significant alterations to the various microbial communities.

Several studies have demonstrated how these pregnancy and delivery factors individually affect the infant microbiome^[3,4,7-9]^, but few have considered a more comprehensive approach. Some studies have accounted for multiple factors; for example, one study found that, after adjusting for maternal race, prenatal antifungal use and intrapartum antibiotic use, maternal GBS status was significantly associated with infant gut bacterial composition at 6 months^[37]^. In another study, maternal obesity was only associated with altered infant gut microbiome composition in vaginally-delivered infants, whereas this association was not seen in Cesarean-delivered infants^[38]^. In this cohort, many of the pregnant individuals with an elevated BMI were also on antibiotics, were GBS-positive, and were not breastfeeding.

Additionally, many of these factors are known to interact with one another, and to make a greater impact on infant health, it may be best to first address overall maternal health before and during pregnancy. For example, although some studies have found an association between antibiotic use in pregnancy and an increased risk of childhood obesity^[39]^, one should also consider the reasons for prenatal antibiotics in the first place. For one, obesity is associated with an increased risk of bacterial infections during pregnancy^[40]^ and, thus, higher rates of antibiotic use^[11]^. Furthermore, the increased risk of childhood obesity may be the result of maternal infection, rather than antibiotic use during pregnancy, as noted by one study^[41]^.

In agreement with previous literature, this study determined that the mode of birth was associated with significant alterations to the baby stool microbiota, including enrichment of aerobic microbes in infants born by Cesarean section. This is unsurprising given the association of vaginal birth with anaerobic vaginal and gut microbes, rather than the Cesarean section seeding of microbes from the skin and external environment^[3,4,42,43]^. The current results contrast with previous findings where antibiotic exposure^[44-46]^ and breastfeeding^[47,48]^ have demonstrated significant association with baby stool microbiota; the lack of detectable signals in this cohort is likely due to the small and phenotypically heterogeneous population.

Numerous alterations to the infant salivary microbiota were associated with gestational age, breastfeeding, and primigravity. While first pregnancy is known to influence labor duration and a recent study suggests its association with altered gut microbiota and increased BMI in children between 1-3 years of age^[49]^, we believe this to be the first instance detailing its influence on the infant’s oral microbiota. At the early timepoint measured in the current study, the oral cavity may be the first bacterial community to exhibit these distinct and novel microbial indicators, which, at a later age, could be consequential to health. Specifically, the changes determined here in oral lactobacilli may have future implications for childhood oral candidiasis and caries development^[50,51]^. The oral cavity also seeds the gut microbiota, and since many systemic diseases are associated with changes in the microbial community of both sites, gaining a better understanding of the factors shaping the oral microbiota could expand our ability to diagnose and predict both oral and systemic diseases^[50]^.

Maternal obesity was significantly associated with variation in only the vaginal microbiota, indicating that it may not be as relevant to the infant microbiota as other birth factors including mode of delivery, primigravity, breastfeeding, and gestational age. Again, it is possible that our limited sample size and heterogeneous maternal population decreased power to detect significant microbiota-related factors, or that infant manifestations of the relevant factors were detectable beyond the postpartum timepoint measured in this study (6-8 weeks after birth)^[52]^. In this regard, the factors determined to be significant microbiota covariates (most of which were investigated in maternal and infant samples from multiple anatomical sites here for the first time) warrant further exploration in larger longitudinal studies to determine the role (if any) they have on microbiota development and overall health later in life^[53]^.

The success of fecal microbiome transplant for microbially-involved and systemic diseases beyond *Clostridioides difficile* infection has led to the proposal of transplanting alternative materials and anatomical regions, including the vaginal microbiome for bacterial vaginosis and urinary tract infection^[54-60]^. Similarly, based on the known negative associations with Cesarean section microbial acquisition, interest in microbiota correction by maternal vaginal seeding has increased in recent years^[43,61-63]^. While this field is still evolving, it is important to characterize the optimally efficacious donor sample; studies diverge in this regard, with some utilizing maternal vaginal material^[43]^, others suggesting maternal gut material^[62]^, or even arguing that species-specific restoration is sufficient^[63]^. While maternal stool was not investigated here, beyond any other birth factor, perinatal period was the most significant driver of vaginal microbiota community structure and function. The baby stool microbiota also shared the fewest microbial taxa with the vaginal sample collected at birth. Thus, if indeed vaginal seeding is to be undertaken, the sample should be collected prior to the onset of labor, after which the microbial community structure and overall function significantly change without recovery by 6 weeks postpartum. The extended displacement of vaginal lactobacilli during and after birth indicates that additional microbial therapeutic support may be advantageous to all perinatal individuals, and not just those with Cesarean births^[64]^. Maternal probiotic supplementation during pregnancy and postpartum has shown promise in long-lasting health outcomes in both mother and child related to obesity^[65,66]^, allergy^[67]^, depression and anxiety^[68]^, and necrotizing enterocolitis^[69]^, among other conditions, warranting consideration for incorporation into standard perinatal care.

This study has several limitations. As previously mentioned, the sample size was modest, and the maternal cohort was heterogeneous in BMI, antibiotic exposure, GBS status, age, delivery, and infant feeding method. Although a multivariate approach was undertaken to account for such differences, a future study with a larger sample size could have better precision in detecting and adjusting for potential confounders. Maternal stool was also not investigated here. This study employed 16S rRNA amplicon sequencing of all samples; this technique provides relative compositional, but not absolute abundance information. It also did not provide taxonomic annotation to the species level in all cases, so caution should be taken when comparing these data and taxonomic annotations in future studies. The 16S rRNA amplicon data were used to infer functional metagenomes with PICRUSt2; although this technique demonstrates accuracy in samples of human origin^[27,70]^, it is not a replacement for whole shotgun metagenomic sequencing which could be employed in the future to validate the current findings.

## CONCLUSION

Overall, this study provides the first look at how a multitude of birth factors may influence maternal and infant microbiota. The maternal salivary and vaginal microbiota were associated most significantly with temporal factors related to birth, while the infant samples were influenced to a lesser degree by a range of factors including mode of delivery, breastfeeding, and gestational age. As obesity, GBS status, and antibiotic use continue to increase in our population^[71]^, it is important to concurrently study these features and their interaction and effect on both the maternal and infant microbiota. Whether these differential features manifest in the infant’s later life disease risk requires additional study.

## DECLARATIONS

### Availability of data and materials

All 16S rRNA gene sequencing data have been deposited into the NCBI Sequence Read Archive, BioProject ID # PRJNA801237.

### Copyright

© The Author(s) 2023.
